# Supplementary material for: Highly parallelized laboratory evolution of wine yeasts for enhanced metabolic phenotypes
Source: Mol Syst Biol. 2024 Aug 22;20(10):2. doi: 10.1038/s44320-024-00059-0 (PMC11450223; doi:10.1038/s44320-024-00059-0)
Supplement: Supplementary file 1 — Appendix [file 44320_2024_59_MOESM1_ESM.pdf]

Appendix for:

Highly parallelized laboratory evolution of wine yeasts for enhanced metabolic phenotypes

Payam Ghiaci, Paula Jouhten, Nikolay Martyushenko, Helena Roca-Mesa, Jennifer Vázquez, Dimitrios Konstantinidis, Simon Stenberg, Sergej Andrejev, Kristina Grkovska, Albert Mas, Gemma Beltran, Eivind Almaas, Kiran R. Patil, Jonas Warringer

Corresponding authors: jonas.warringer@cmb.gu.se, eivind.almaas@ntnu.no, kp533@cam.ac.uk

Table of contents:

Appendix Table S1	page 2-7
Appendix Table S2	page 8-11
Appendix Table S3	page 12-14
Appendix Table S4	page 15
Appendix Table S5	page 16-17
Appendix Table S6	page 18
Appendix Figure S1	page 19
Appendix Figure S2	page 20
Appendix Figure S3	page 21
Appendix Figure S4	page 22
Appendix Figure S5	page 23
Appendix Figure S6	page 24

Appendix Table S1. Strains used in the study. Wine yeasts = commercial wine yeasts owned or marketed by Lallemand Inc (Canada). Vineyard yeasts = natural, non-commercialized vineyard yeasts; from grapes or vineyard soil in the DOQ Priorat wine-making region in Catalonia, and identified as *S. cerevisiae* using restriction fragment length polymorphisms.

Name	Labelled as	Type	Description	Heterozygosity ratio[a]
L 71B	E1	Commercial wine yeasts	Lalvin 71B®. Isolated by INRA-Narbone (France)	0.10
L CLOS	E2	Commercial wine yeasts	Lalvin CLOS®. Isolated by URV in DOQ Priorat wine region (Spain)	0.10
L QA23	E3	Commercial wine yeasts	Lalvin QA23®. Isolated by UTAD in Vinhos verdes wine region (Portugal). *S. cerevisiae bayanus*	0.25
LEC	E4	Commercial wine yeasts	Lalvin EC1118®. Isolated in Champagne wine region (France). *S. cerevisiae bayanus*	0.52
L T73C	E5	Commercial wine yeasts	Lalvin T73®. Isolated by IATA-CSIC in DO Alicante wine region (Spain). *S. cerevisiae bayanus*	0.06
U VN	E6	Commercial wine yeasts	Uvaferm VN®. Isolated by IVICAM (Spain) in DO La Mancha wine region (Spain)	0.25
U BC	E7	Commercial wine yeasts	Uvaferm BC®. Isolated by Institute Pasteur	0.03

			(France). *S. cerevisiae bayanus*	
U BDX	E8	Commercial wine yeasts	Uvaferm BDX®. Isolated by U. Bordeaux (France)	0.10
U CS2	E9 (Ctrl strain)	Commercial wine yeasts	Uvaferm CS2®. Used as the control strain throughout the phenotyping experiments.	0.66
U EXE	E10	Commercial wine yeasts	Uvaferm EXENCE®. Isolated by IWB in Stellenbosch (South Africa), result of the crossing of two Sc strains.	0.57
U WAM	E11	Commercial wine yeasts	Uvaferm WAM®. Isolated by U. Valladolid in DO Rueda wine region (Spain).	0.03
U 43	E12	Commercial wine yeasts	Uvaferm 43®. Isolated by Institute Inter Rhône (France). Fructofilic yeast. *S. cerevisiae bayanus*	0.28
U CEG	D12	Commercial wine yeasts	Uvaferm CEG®. Isolated by Geisenheim Research Station (Germany)	0.30
V BMW58	D11	Commercial wine yeasts	Velluto BMV58®. Isolated by IATA-CSIC in DO Valencia wine region (Spain). *S. uvarum*	ND

Cross Evolution	D10	Commercial wine yeasts	Cross Evolution®. Selected by IWB in Stellenbosch (South Africa), result of the backcrossing of two Sc strains.	0.33
SL6	G1	Local cellar isolates	Natural isolate from DOQ Priorat wine region (Ferrer-Bobet winery)	0.03
SFB2	G2	Local cellar isolates	Natural isolate from DOQ Priorat wine region (Ferrer-Bobet winery)	0.04
SFB1	G3	Local cellar isolates	Natural isolate from DOQ Priorat wine region (Ferrer-Bobet winery)	0.03
SFB3	G4	Local cellar isolates	Natural isolate from DOQ Priorat wine region (Ferrer-Bobet winery)	0.10
SFB5	G5	Local cellar isolates	Natural isolate from DOQ Priorat wine region (Ferrer-Bobet winery)	0.04
SFB4	G6	Local cellar isolates	Natural isolate from DOQ Priorat wine region (Ferrer-Bobet winery)	0.03
SFB7	G7	Local cellar isolates	Natural isolate from DOQ Priorat wine region (Ferrer-Bobet winery)	0.04
SFB6	G8	Local cellar isolates	Natural isolate from DOQ Priorat wine region (Ferrer-Bobet winery)	0.03

SFB10	G9	Local cellar isolates	Natural isolate from DOQ Priorat wine region (Ferrer-Bobet winery)	0.26
SFB9	G10	Local cellar isolates	Natural isolate from DOQ Priorat wine region (Ferrer-Bobet winery)	0.22
SFB8	G11	Local cellar isolates	Natural isolate from DOQ Priorat wine region (Ferrer-Bobet winery)	0.04
SL4	G12	Local cellar isolates	Natural isolate from DOQ Priorat wine region (Ferrer-Bobet winery)	0.04
SL3	F12	Local cellar isolates	Natural isolate from DOQ Priorat wine region (Ferrer-Bobet winery)	0.02
M2	M2	Local cellar isolates	Natural isolate from DOQ Priorat wine region (Mas Perinet winery)	0.14
M3	M3	Local cellar isolates	Natural isolate from DOQ Priorat wine region (Mas Perinet winery)	0.11
M4	M4	Local cellar isolates	Natural isolate from DOQ Priorat wine region (Mas Perinet winery)	0.26
M5	M5	Local cellar isolates	Natural isolate from DOQ Priorat wine region (Mas Perinet winery)	0.03
M6	M6	Local cellar isolates	Natural isolate from DOQ Priorat wine region (Mas Perinet winery)	0.03

M7	M7	Local cellar isolates	Natural isolate from DOQ Priorat wine region (Mas Perinet winery)	0.05
M9	M9	Local cellar isolates	Natural isolate from DOQ Priorat wine region (Mas Perinet winery)	0.03
M10	M10	Local cellar isolates	Natural isolate from DOQ Priorat wine region (Mas Perinet winery)	0.04
M11	M11	Local cellar isolates	Natural isolate from DOQ Priorat wine region (Mas Perinet winery)	0.19
M12	M12	Local cellar isolates	Natural isolate from DOQ Priorat wine region (Mas Perinet winery)	0.67
M13	M13	Local cellar isolates	Natural isolate from DOQ Priorat wine region (Mas Perinet winery)	0.12
T3	T3	Local cellar isolates	Natural isolate from DO Terra Alta wine region	0.04
T4	T4	Local cellar isolates	Natural isolate from DO Terra Alta wine region	0.03
T5	T5	Local cellar isolates	Natural isolate from DO Terra Alta wine region	0.03
T6	T6	Local cellar isolates	Natural isolate from DO Terra Alta wine region	0.03
T7	T7	Local cellar isolates	Natural isolate from DO Terra Alta wine region	0.03

T8	T8	Local cellar isolates	Natural isolate from DO Terra Alta wine region	0.03
T14	T14	Local cellar isolates	Natural isolate from DO Terra Alta wine region	0.05
T15	T15	Local cellar isolates	Natural isolate from DO Terra Alta wine region	0.03
T19	T19	Local cellar isolates	Natural isolate from DO Terra Alta wine region	0.02

[a] The heterozygosity ratio was calculated as the number of heterozygotic SNPs/ the number of homozygotic SNPs.

Appendix Table S2. Description and rationale of ALE selection environments used in the study.

ALE selection	Background	Description & Rationale
NCR relaxation	*S. cerevisiae* use of organic nitrogen sources common in grape must (proline, arginine) is often repressed by the presence of inorganic ammonium. Ammonium stimulates the nitrogen catabolite repression system (NCR), which transcriptionally and post-transcriptionally represses genes required for uptake and catabolism of e.g. proline and arginine. Incomplete use of these nitrogen sources leads to stuck fermentations, inability to consume all sugar and invasion of the must by spoiling microorganisms[107]. Amines (RCH_2NH_2), such as methylamine, likewise stimulates NCR and represses proline and arginine use. *Saccharomyces cerevisiae* lacks the amine oxidases *AMO1* and *AMO2* and is therefore not capable of using methylated amines as nitrogen sources[108, 109]. Methylamine therefore suppresses the use of proline and arginine by activating NCR but does provide the nitrogen that yeast requires for growth.	Select evolving populations on a synthetic grape must medium that only contains arginine and proline as nitrogen sources (15 mg/L each) and where use of arginine and proline is heavily repressed by the presence of methylamine (10 g/L). We expect a subset of populations to adapt by reducing the activation of NCR by methylamine, allowing yeast to better use arginine and proline to support growth. Because of the interconnectivity of intracellular nitrogen metabolite pools, we also expect increased flow through the proline and arginine catabolism systems to spill over into higher flows through other nitrogen metabolic pathways, such as those involving branched chain amino acids. This may result in increased production of aroma compounds, and enhanced wine taste and smell.

GR relaxation	*S. cerevisiae* often use fructose in grape must less well in the presence of glucose. Incomplete use of fructose often leads to stuck fermentations, inability to consume all sugar and invasion of the must by spoiling microorganisms[110]. 2-deoxy-D-glucose, which has the 2-hydroxyl group replaced by hydrogen, possess many of the properties of glucose, but because it competitively inhibits the production of glucose-6-phosphate[111], it cannot undergo glycolysis and cannot be used carbon or energy source by *Saccharomyces cerevisiae.*	Select evolving populations on a synthetic grape must medium that only contains fructose as carbon source and where use of fructose is disfavoured due to the presence of 2-deoxy-D-glucose (2g/L). We expect a subset of populations to adapt by relaxation of glucose repression, allowing earlier and faster use of fructose. Such strains will be particularly useful for re-starting fermentations that have stuck due to incomplete use of fructose.
Glutathione production	Glutathione prevents oxidation of wine but is rarely present in notable concentrations in grape must. Yeast produces glutathione from glutamate, cysteine and glycine in a two-step reaction catalyzed by *GSH1* and *GSH2* and can be excreted[112]. The intracellular demand for, and production of glutathione, increases heavily under oxidizing conditions imposed by addition of the oxidizing agent diamide[113].	Select evolving populations on a synthetic grape must medium only containing glutamate, cysteine and glycine (equal proportions; standard amounts of nitrogen retained 140 mg N/L) as nitrogen sources, increasing their intracellular pools and the flow through glutathione biosynthesis. Diamide is added at growth limiting concentrations (1.5 mM) to simultaneously increase demand for high intracellular glutathione levels. We expect a subset of populations to adapt by mutations that directly (e.g. glutathione biosynthesis) or indirectly (e.g. precursor uptake) increase net production of glutathione.

Aroma production	Wine aroma compounds, e.g. fusel alcohols, produced by wine yeasts are bio-synthesized via pathways, e.g. the Ehrlich pathway[114], originating in the intracellular pools of branched chain amino acids, such as isoleucine, phenylalanine and valine.	Select evolving populations on a synthetic grape must medium that only contains isoleucine, phenylalanine and valine (equal proportions; standard amount of nitrogen retained) as nitrogen sources. We expect a subset of populations to adapt by mutations that directly (e.g. the Ehrlich pathway) or indirectly (e.g. BCAA uptake) increases the production of fusel alcohols.
Ethanol tolerance	Due to climate change, the sugar content of grapes creeps upwards. Because, yeast ferments sugar to ethanol, so do the ethanol content of wine. To avoid stuck fermentations, sugar left in the wine, and wine spoilage, this demands wine yeast capable of growth and fermentation at high concentrations of ethanol. The need is particularly pronounced for sparkling wine, which is produced by two serial fermentations of grape must. The second fermentation is initiated by inoculating yeast into wine already high in ethanol content, exposing the yeast to an ethanol shock unless it is pre-adapted [115]. Ethanol is volatile and evaporates under open environment selection experiments. Less volatile alcohols, such as 1-butanol (bp 118 C vs. 78 C of EtOH), have cellular effects that mimic those of ethanol, and strains	Select evolving populations on a synthetic grape must medium supplemented by growth limiting (1.3 % v/v) concentrations of 1-butanol. We expect a subset of populations to adapt by increasing their tolerance to alcohols in general, including to ethanol.

	resistant to 1-butanol tend to be resistant to ethanol.	
Sugar tolerance	Due to climate change, the sugar content of grapes creeps upwards[116]. High sugar content exposes yeast to osmotic and ethanol stress and affects sugar uptake by emphasizing the importance of low affinity hexose transporters.	Select evolving populations on a synthetic grape must medium with high (35 %; standard proportions of glucose and fructose retained) sugar concentrations. We expect some populations to adapt by increasing their tolerance to osmotic and ethanol stress, and to be able to take up sugar faster and more efficiently at higher sugar concentrations.
Vitamin starvation	Vitamins are required for yeast growth and metabolism because of their roles as enzymatic cofactors. In many grape musts, access to vitamins constrains yeast growth and metabolism lead to stuck fermentation, sugar and nitrogen left in the medium, and wine spoilage[107]. The problem is exacerbated by climate change, which leads to faster grape maturation and lower vitamin content of mature grapes[117].	Select evolving populations on a synthetic grape must medium poor in vitamins (1 % of normal; standard proportions retained). We expect some populations to adapt by increasing their capacity to grow and metabolize at low vitamin concentrations.
Nitrogen starvation	Grape must is often rich in carbon, but poor in nitrogen, leading to nitrogen starved yeast, stuck fermentations, sugar left in the wine and wine spoilage[118].	Select evolving populations on a synthetic grape must medium poor in nitrogen (10 % of normal; standard proportions retained). We expect some populations to adapt by increasing their capacity to grow and metabolize at low nitrogen concentrations.

Appendix Table S3. Growth medium composition for each ALE selection and side-effect environments. Synthetic grape must[34] was the background media throughout all the experiments.

Abbreviation	Description	ALE selection	Side-effect environment
S-NCR	SGM adjusted as [arginine (15 mg N/l) + proline (15 mg N/l)] as sole N sources + 1% Methyl amine	NCR relaxation	
S-GR	SGM adjusted as 20% Fru. + 2-deoxy glucose (0.2 g/100 ml)	GR relaxation	
S-Gluth	SGM adjusted as [Glycine (47 mg N/l) + Glutamine (47 mg N/l) + Cysteine (47 mg N/l)] as sole N source + 1.5 mM Diamide	Glutathione production	
S-Aroma	SGM adjusted as [Valine (10 mg N/l) + Iso-leucine (10 mg N/l) + Phenylalanine (10 mg N/l)] as sole N sources	Aroma production	
S-But	SGM + 1.3% 1-butanol (v/v)	Ethanol tolerance	
S-35Sug	SGM adjusted as 17.5% Glu. + 17.5% Fru as C source	Sugar tolerance	
S-Vit	SGM adjusted as 1% of normal vitamin concentration	Vitamin starvation	
S-Nit	SGM adjusted as 10% of normal amino acid concentration	Nitrogen starvation	
S-40Sug	SGM adjusted as 20% Glu. + 20% Fru as C source	---	C utilization
S-Fru	SGM adjusted as 20% Fructose as a C source	---	C utilization

S-Lac	SGM adjusted as 2% Lactose as a C source	---	C utilization
S-Mal	SGM adjusted as 2% Maltose as a C source	---	C utilization
S-Man	SGM adjusted as 2% Mannose as a C source	---	C utilization
S-Raf	SGM adjusted as 2% Raffinose as a C source	---	C utilization
S-Gly	SGM adjusted as 2% Glycerol as a C source	---	C utilization
S-Gal	SGM adjusted as 2% Galactose as a C source	---	C utilization
S-Ala	SGM adjusted as Alanine (mg N/l) as sole N source	---	N utilization
S-Asp	SGM adjusted as Aspartic acid (mg N/l) as sole N source	---	N utilization
S-Glt	SGM adjusted as Glutamine (mg N/l) as sole N source	---	N utilization
S-Glyc	SGM adjusted as Glycine (mg N/l) as sole N source	---	N utilization
S-GABA	SGM adjusted as GABA (mg N/l) as sole N source	---	N utilization
S-Met	SGM adjusted as Methionine (mg N/l) as sole N source	---	N utilization
S-Pro	SGM adjusted as Proline (mg N/l) as sole N source	---	N utilization
S-Phe	SGM adjusted as Phenylalanine (mg N/l) as sole N source	---	N utilization

S-Urea	SGM adjusted as Urea (mg N/l) as sole N source	---	N utilization
S-Val	SGM adjusted as Valine (mg N/l) as sole N source	---	N utilization

Appendix Table S4. List of strains used in validations at semi-industrial fermentation of grape musts.

Parental lineage	ALE evolved population	ALE selection environment
M3	M3 (2,35)	High Sugar
E2	E2 (26,37)	High Sugar
G11	G11 (23,22)	High Sugar
M12	M12 (18,23)	High Sugar
E9	E9 (0,7)	High Ethanol

Appendix Table S5. ALE evolved populations for which glutathione levels were measured.

Parental lineage	ALE population evolved for improved glutathione use
E6	E6 (30,7)
	E6 (31,30)
G10	G10 (19,18)
T3	T3 (15,35)
	T3 (15,32)
T5	T5 (11,15)
T4	T4 (0,43)
	T4 (0,19)
E11	E11 (22,31)
G9	G9 (13,1)
T15	T15 (10,19)
M3	M3 (1,35)
G6	G6 (1,36)

M4	M4 (15,28)
E7	E7 (15,46)
E5	E5 (15,39)
M7	M7 (9,24)
D12	D12 (21,34)

Appendix Table S6. List and description of sequenced ALE populations

Name (x, y position on solid media)	Selection environment
T3 (15,35)	Glutathione production
T3 (15,32)	Glutathione production
M4 (15,28)	Glutathione production
M7 (9,24)	Glutathione production
M9 (21,0)	High Sugar
E2 (26,37)	High Sugar
F12 (6,31)	High Sugar
G1 (30,13)	High Sugar
G10 (16,17)	High Sugar
M3 (0,33)	High Sugar
M3 (2,35)	High Sugar
M3 (1,34)	High Sugar
T14 (16,15)	High Sugar
E11 (21,4)	NCR relaxation
G6 (0,37)	NCR relaxation
M4 (14,5)	NCR relaxation
M7 (11,24)	NCR relaxation
T4 (3,41)	NCR relaxation
T6 (5,10)	NCR relaxation
M5 (4,43)	NCR relaxation
E9 (0,7)	High EtOH
G7 (30,45)	High EtOH
M2 (13,40)	High EtOH
T3 (12,35)	High EtOH
T3 (13,35)	High EtOH
T3 (15,35)	High EtOH

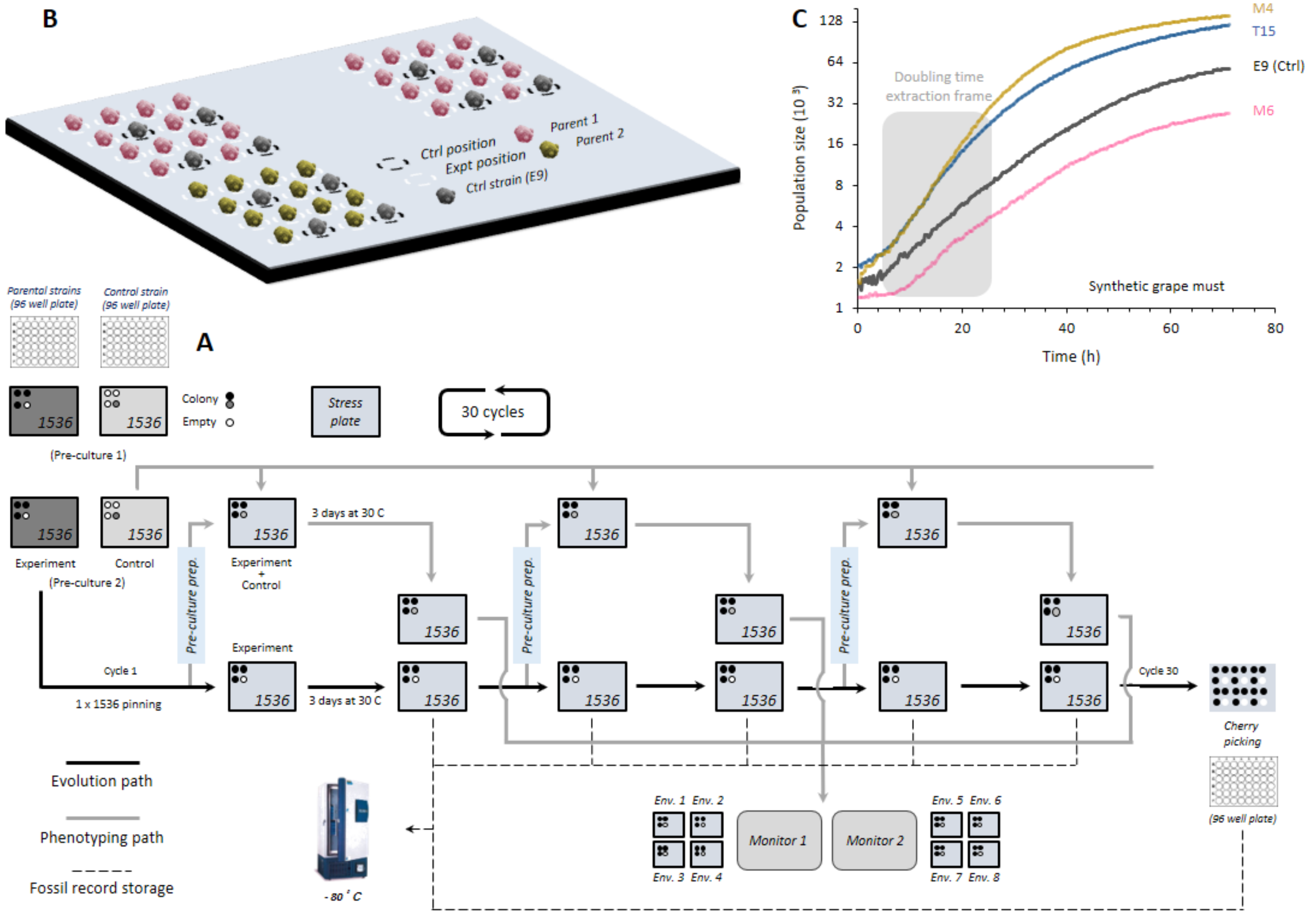

Appendix Figure S1. Design of wine yeast ALE experiments. (A) 48 commercial and non-commercial wine yeast strain (Supplementary Table 1) were ALE evolved as replicated (*n=24*) asexual populations over 30 growth cycles. ALE populations were cultivated (black arrows = evolution track) as colonies on eight synthetic grape media designed to select for traits desired by the wine industry. We generated 48 parent populations from single clones of each parental lineage (not shown) and replicated these 24x to generate a 1152 colony array cultivated on synthetic grape must. Colonies were stored at -80ºC in 96 arrays in 20% glycerol. Frozen stocks were revived on synthetic grape must and transferred to evolution plates representing the eight selection regimes. The 9216 ALE populations were passed through 30 cycles of growth, sampling and transfer (n=~10^5 cells) to fresh plates. We stored the cycle 30 end-point of each population as a frozen ALE record before reviving and cultivating these in 1536 format, while counting cells in each growing colony (grey arrows=phenotyping track). The 1152 cycle 0 start points were revived and cultivated in parallel, on separate plates. Populations were cultivated as multiple replicates (*n*=2-4; on separate plates) in each of the eight designed selection environments and in the 18 nitrogen or carbon limited side effect environments. A fixed control was introduced and cultivated in every 4th position on every plate and used to control for systematic growth variation between and within plates. We extracted cell-doubling times from high quality growth curves, log_2 transformed and normalized these measures to those of the 384 fixed controls on the same plate. **(B)** A zoom-in view of one experimental plate, showing the arrangement of experimental and fixed control populations. **(C)** Growth of wine yeasts (color) growing on synthetic grape must. Black = fixed control (parental lineage E9). Grey field: time window in which the cell doubling time was extracted.

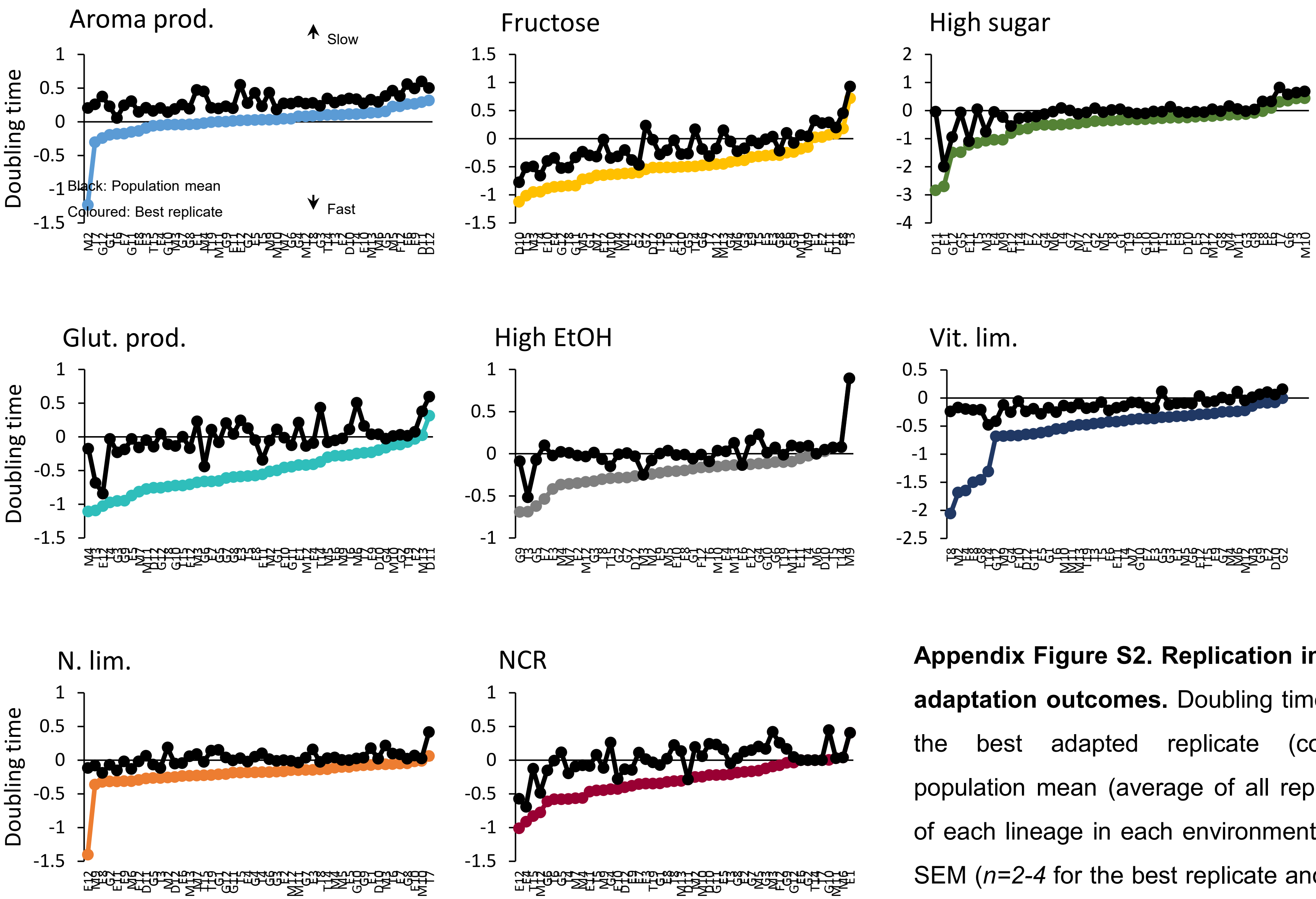

Appendix Figure S2. Replication improves ALE adaptation outcomes. Doubling time line chart of the best adapted replicate (coloured) and population mean (average of all replicates: black) of each lineage in each environment. Error bars = SEM (*n=2-4* for the best replicate and *n=24* for the population).

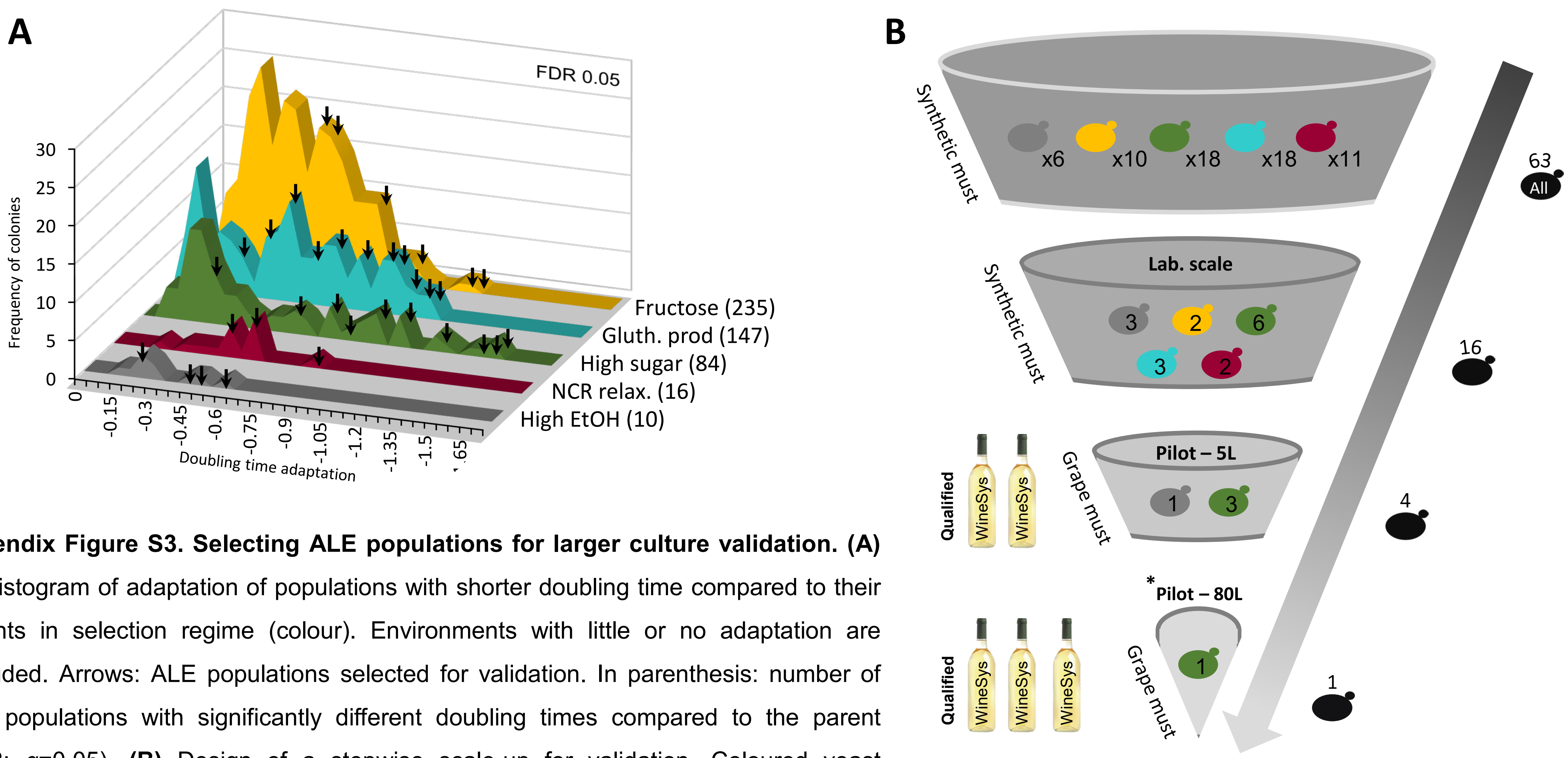

Appendix Figure S3. Selecting ALE populations for larger culture validation. (A) 3D histogram of adaptation of populations with shorter doubling time compared to their parents in selection regime (colour). Environments with little or no adaptation are excluded. Arrows: ALE populations selected for validation. In parenthesis: number of ALE populations with significantly different doubling times compared to the parent (FDR: *q*=0.05). **(B)** Design of a stepwise scale-up for validation. Coloured yeast represents ALE selection environments. Numbers = number of populations tested.

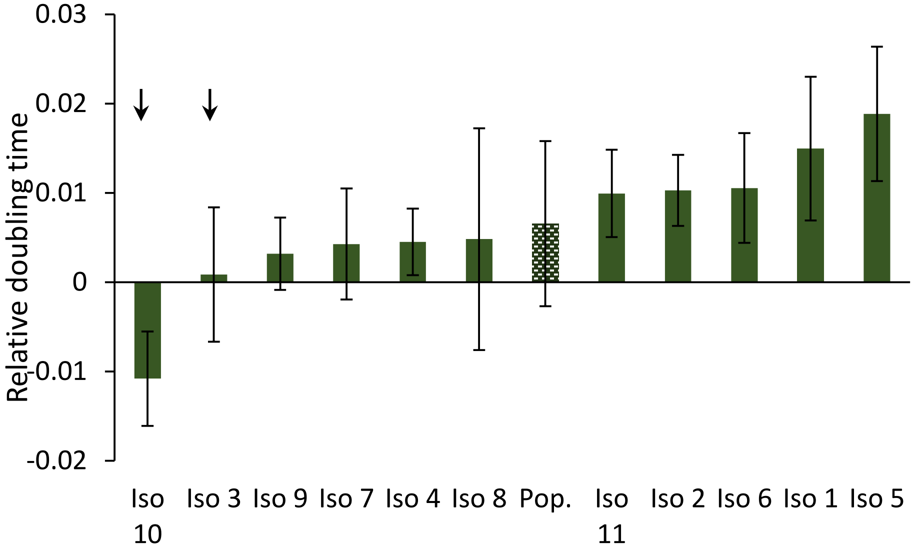

Appendix Figure S4. Selecting clones for 80L scale-up experiments. 11 clones (iso 1-11) were isolated from the high sugar adapted ALE population E2 (26, 37) (pop.) and clonally expanded (n=24) on synthetic grape must. Their log_2 doubling time normalized to that of the global control strain E2 is shown and compared to that of ALE population E2 (26, 37). Arrows: $p<0.05$ (one sided Student's t-test), error bars = S. d. Isolates 3 and 10 were selected for scale-up experiments in 80L grape must.

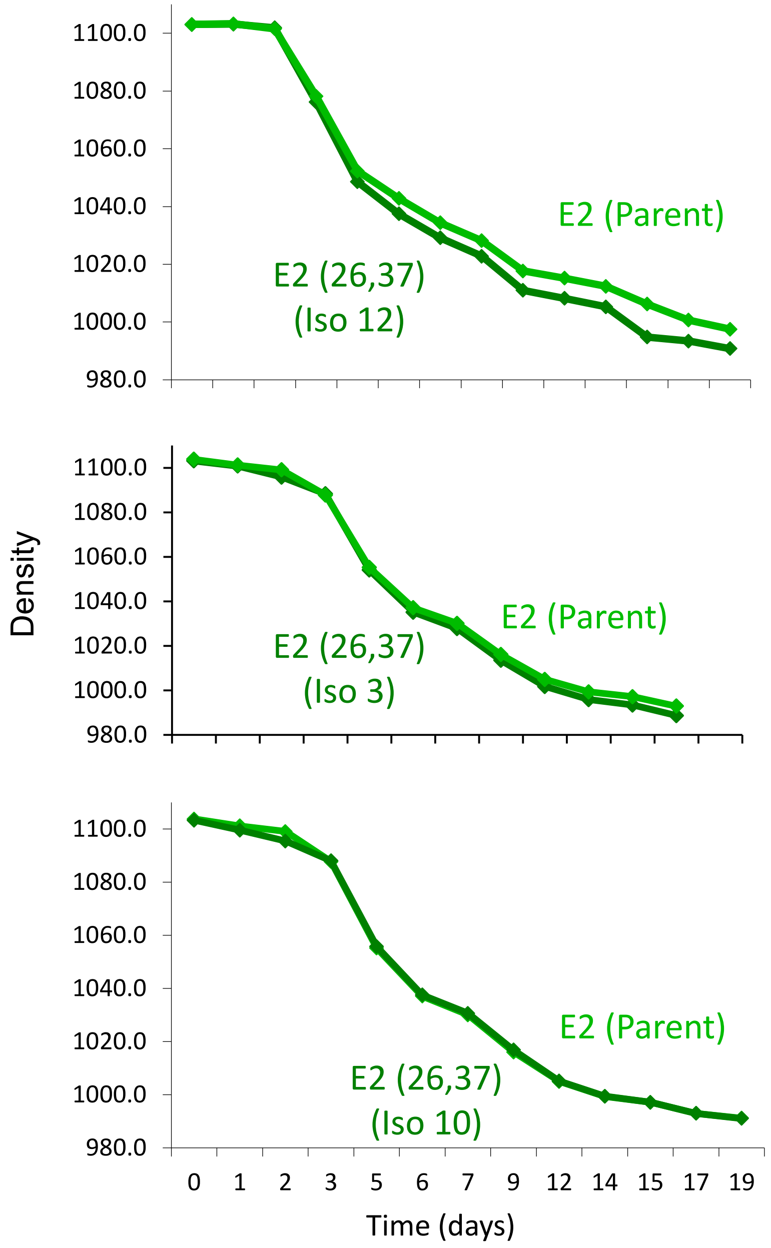

Appendix Figure S5. High sugar adapted ALE clonal isolates ferment grape must well in larger liquid cultures. We followed the capacity of clonal isolates from the high sugar adapted ALE population E2 (26, 37) and the parent E2 strain to ferment grape must in 80L cultures, by measuring the grape must density. The time-resolved grape must density is shown for isolates (iso) 3 and 12 and the corresponding parent strain cultivated in the same grape must.

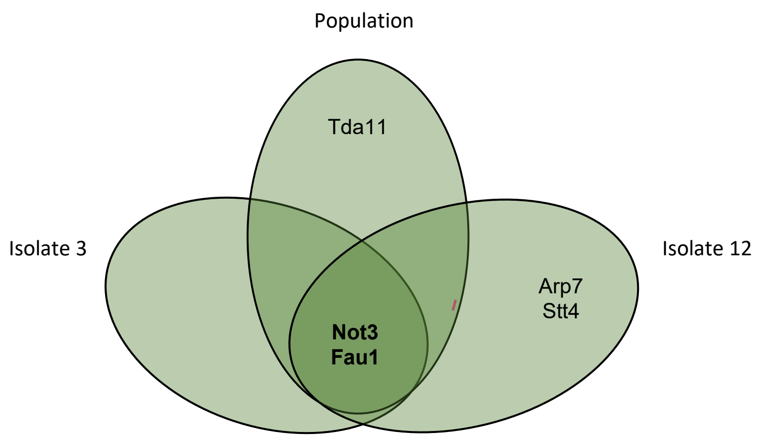

Appendix Figure S6. Sequence variations in high sugar adapted ALE clones. We sequenced the high sugar adapted ALE population E2 (26, 37) as well as isolates 3 and 12 from this population and called variants relative the E2 parent strain. The Venn diagram shows the genes containing called single nucleotide variants in the clones and in the high sugar adapted ALE population E2 (26, 37)..
