# Supplementary material for: Highly parallelized laboratory evolution of wine yeasts for enhanced metabolic phenotypes
Source: Mol Syst Biol. 2024 Aug 22;20(10):2. doi: 10.1038/s44320-024-00059-0 (PMC11450223; doi:10.1038/s44320-024-00059-0)
Supplement: Supplementary file 11 — Expanded View Figures [file 44320_2024_59_MOESM11_ESM.pdf]

Expanded View Figures

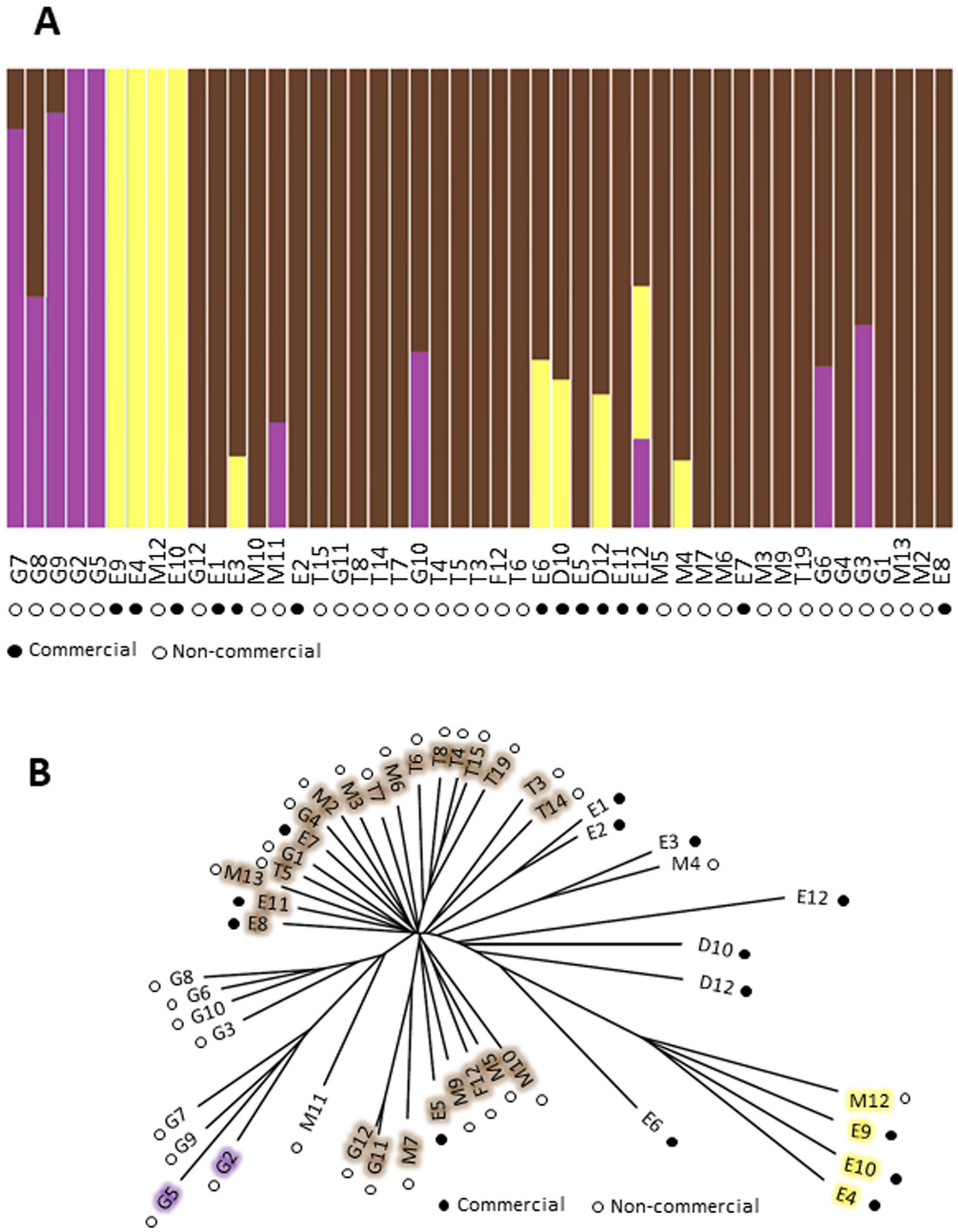

Figure EV1. Population structure of parent wine strains, before ALE.

(**A**) We sequenced the genome of 47 commercial (•) and noncommercial (○) euploid diploids wine yeast lineages (Appendix Table S1) using Illumina and called 42,599 SNPs against the Lalvin EC1118® wine strain reference genome. We varied the number of populations (K) between 1 and 10 and show the population structure and admixture for $K = 4$ (color). (**B**) Phylogeny (neighbor-joining tree) of 47 wine yeast lineages. Text color = non-admixed genetic groups identified by the admixture mapping in (**A**). Admixed strains are left uncolored. Bar = SNP distance.

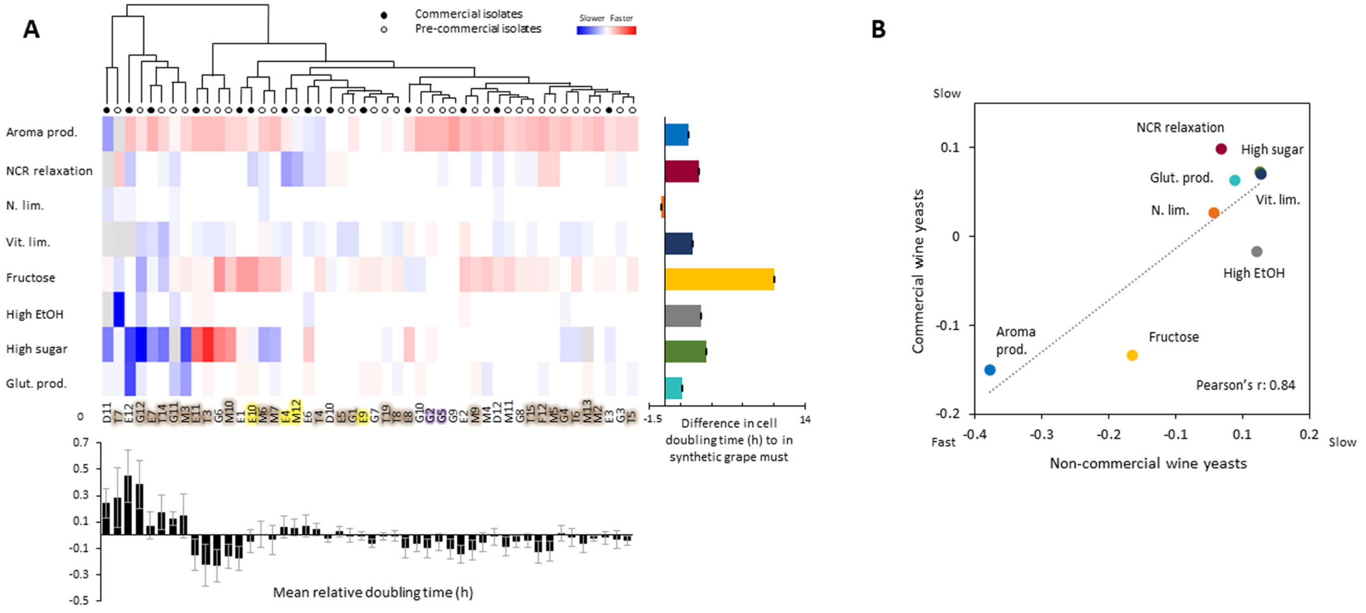

Figure EV2. Strength of ALE selection.

Cell doubling times of wine yeasts in selection environments. (**A**) *Central panel (heatmap):* Cell doubling times (mean: $n = 24$ replicate ALE populations) of each wine yeast normalized to the fixed control (color). No color: fixed control. Wine yeast names are colored based on population (see Fig. EV1). *Upper panel:* Hierarchical clustering of wine yeast based on similarity in cell doubling times, using Pearson's r and strain averages (for groups). *Left panel*: Variance across wine yeasts ($n = 48$ strains) in mean cell doubling time (h). *Right panel*: Strength of selection, estimated as mean ($n = 48$ strains) difference (h) in cell doubling time in an environment, as compared to in synthetic grape must (SGM). Error bars = SEM. *Bottom panel*: Some wine yeasts are general slow growers, reflecting limited adaptation to synthetic grape must. Mean of normalized cell doubling times for each wine yeast, across all selection environments. Error bars = SEM ($n = 8$ environments). (**B**) Commercial ($n = 15$ strains) and noncommercial ($n = 33$ strains) wine yeast grow equally well in all selection environments. Mean cell doubling times normalized to the fixed control are shown. Broken line = linear regression. Source data are available online for this figure.

 © The Author(s)

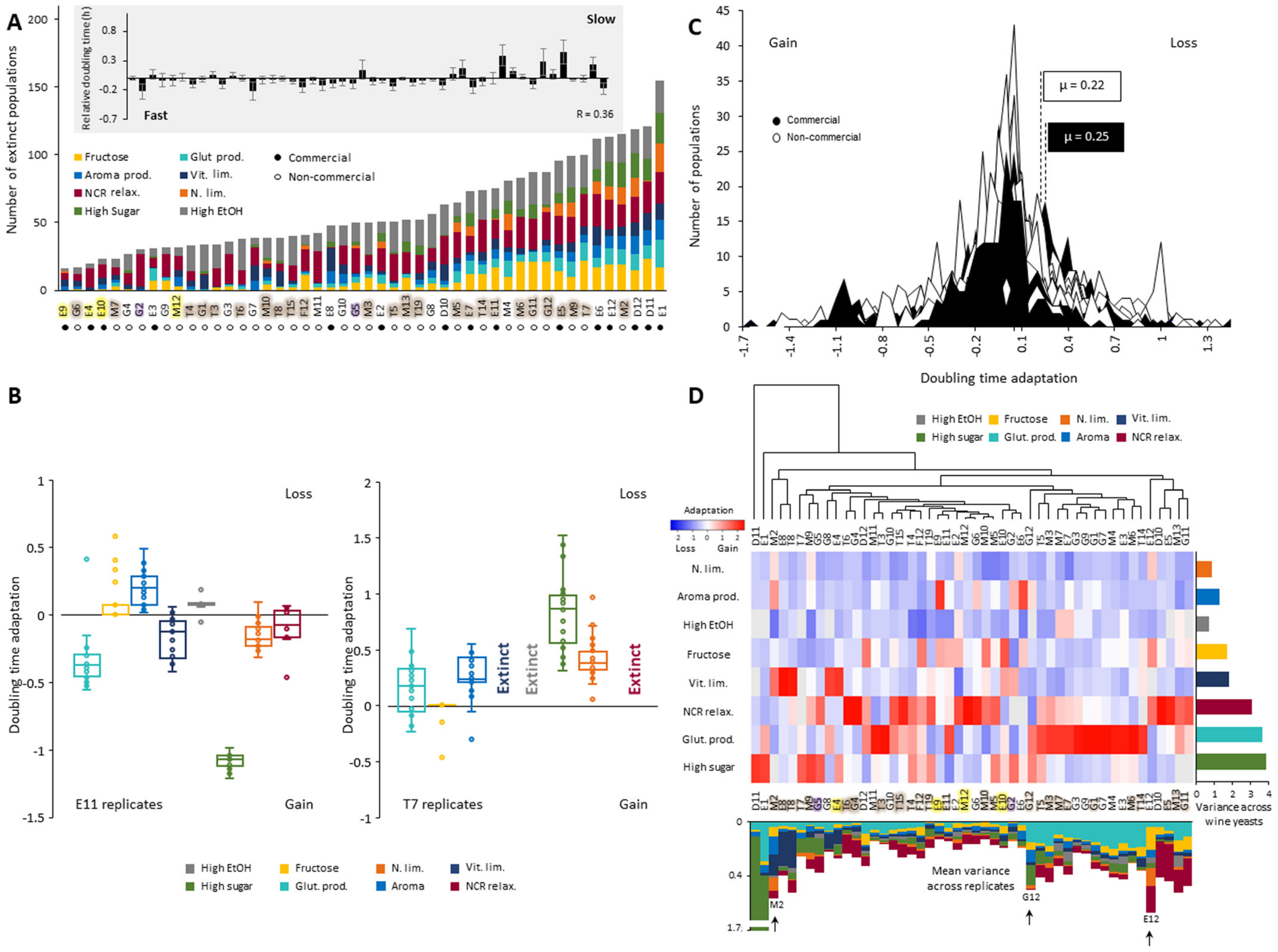

Figure EV3. Adaptation of ALE wine yeasts.

(**A**) Extinct populations: stacked bar plot of number of extinct populations, in each environment. Inset: mean of (log_2, normalized) cell doubling time for each wine yeast ($n = 24$ replicate ALE populations, extinct populations excluded, in each environment) across all environments ($n = 8$ environments). Error bars: SEM ($n = 8$). (**B**) Adaptation ($n = 24$ replicate ALE populations, extinct populations excluded) of wine yeasts E11 (left panel) and T7 (right panel) in selection environments (color). Box: interquartile range, line: median: whiskers 1.5x interquartile range, outliers: populations outside interquartile range. (**C**) Histogram of adaptation for all commercial (black, *n* = *2880 ALE populations)* and noncommercial (white, *n* = *6336 ALE populations*) ALE populations, totaled across all selection regimes and wine yeast lineages. Extinct populations are excluded. (**D**) Adaptability has a genotype-by-environment component. *Central panel (heatmap):* Adaptation (mean: $n = 24$ replicate ALE populations) of each wine yeast. No color: no adaptation. Wine yeast names are colored based on population (see Fig. EV1). *Upper panel:* Hierarchical clustering of wine yeast (Pearson's *r*, averages used to cluster groups) based on similarity in adaptation across eight selection regimes. *Right panel*: Variance in mean adaptation between wine yeasts, in each environment. *Bottom panel*: Variance in adaptation between replicated populations ($n = 24$ replicate ALE populations), for each wine yeast in each environment. Lineages mentioned in text are indicated (arrows). Source data are available online for this figure.

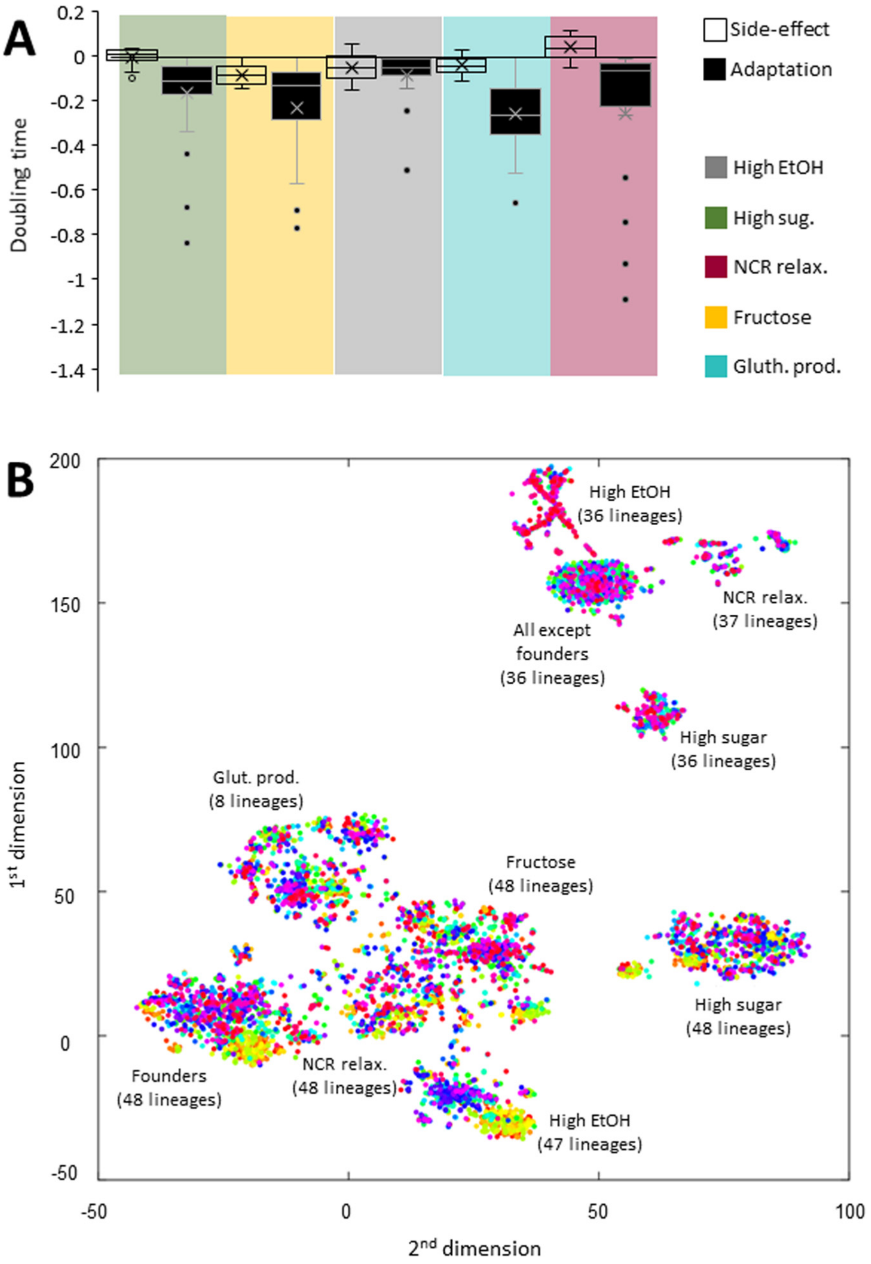

Figure EV4. Side effects evolved by ALE wine yeasts.

(**A**) Side effects (top box) of evolution are smaller than adaptations (bottom box), in all environments. Data across all side effects ($n = 18$ environments), and across all populations ($n = 24$ replicate ALE populations) of all lineages ($n = 48$ strains) in each ALE environment are shown. Box: interquartile range, line: median: whiskers 1.5× interquartile range, outliers: populations outside interquartile range. (**B**) t-Distributed Stochastic Neighbor Embedding (t-SNE) clustering reducing the variance in side effects to two dimensions (*x*, *y*-axes). Each dot represents one population of one lineage in one selection environment, or one starting population. The clustering is identical to that in Fig. 4, but color here indicates lineage. For large clusters, the selection regime and the number of lineages in the cluster are indicated. Note: side effects do not cluster by lineage and each cluster contains representatives of almost all lineages. Source data are available online for this figure.

 © The Author(s)

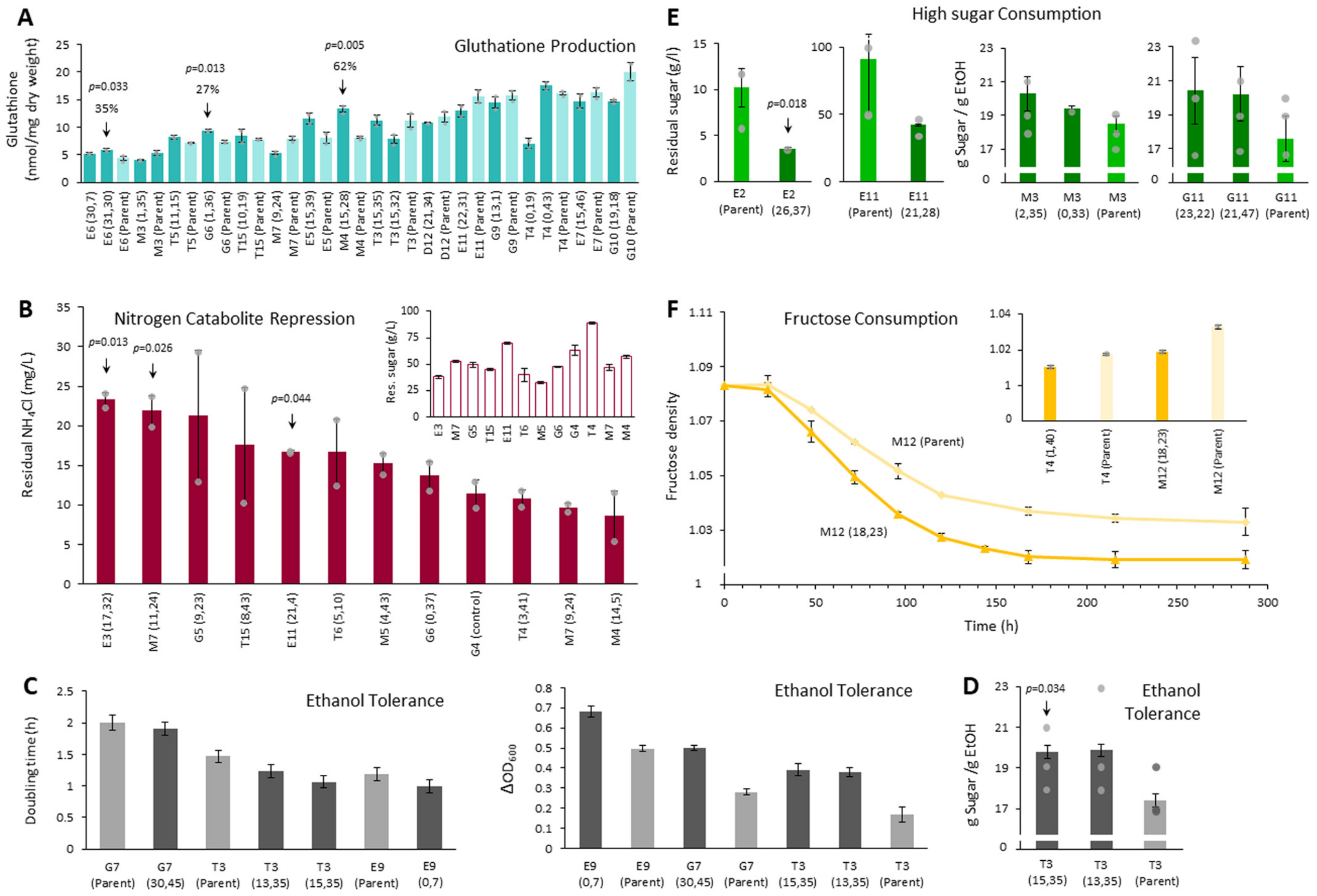

Figure EV5. ALE wine yeasts retain adaptations in more industrial cultures.

(**A**) Eighteen ALE populations selected for higher glutathione production. Arrows: populations producing more glutathione (one-sided Student's t test, $P < 0.05$) than their parental lineages in larger, liquid cultures. Total glutathione (nmol/mg dry weight biomass) at the end of a growth batch cycle (168 h) is shown. Error bars: SEM ($n = 2$ biological replicates). (**B**) Eleven ALE populations with improved growth in the NCR relaxation selection regime were cultivated in liquid synthetic grape must cultures (250 mL) with ammonium (43 mg N at start) as preferred nitrogen source and the residual ammonium was measured at the end of fermentation (360 h). The parental lineage G4, as the best-performing noncommercial wine strain, is shown as reference. Error bars: SEM ($n = 3$ biological replicates). Arrows indicate ALE populations selected for follow-up experiments. Note that non-ammonium nitrogen (total: 100 mg N at start) were present as arginine and proline. *Inset:* residual sugar bar plot (**C**) Six ALE populations, evolved for higher ethanol tolerance, and their parental lineages, were cultivated in low-volume liquid cultures (Bioscreen Inc.) in the presence of 8% ethanol. We tracked their growth continuously and extracted cell doubling times and cell yields. Error bars: SEM ($n = 3$ biological replicates). (**D**) We cultivated two ALE populations evolved for higher ethanol tolerance and expressing this trait in liquid cultures (see Fig. EV5C) in 50 mL synthetic grape must ($n = 2$–3 biological replicates) and compared their fermentation efficiency, measured as the gram sugar consumed per ethanol produced, to that of their parental lineage. Error bars: SEM. Arrow: one-sided Student's *t* test ($P < 0.05$) (**E**) 18 ALE populations, evolved for better growth in high-sugar concentrations, were cultivated in 40 mL liquid high-sugar grape must. We tracked the sugar consumption and ethanol production in each. Two ALE populations showed faster sugar uptake (*top panel*) than their parental lineages and four showed more efficient fermentation (*bottom panel*; gram ethanol produced per gram consumed). Error bars: SEM ($n = 3$ biological replicates) Arrow: significantly different (one-sided Student's *t* test, $P < 0.05$). (**F**) Top ALE populations T4 (1,40) and M12 (18,23), evolved for better fructose use, were cultivated in 40 mL liquid fructose containing synthetic grape must in the presence of the glucose analog 2-deoxyglucose. We compared their sugar fructose consumption to that of the parental lineages. Error bars: SEM ($n = 3$ biological replicates). *Inset:* end fructose density of two tested populations ($P = 0.05$). The line plot shows density kinetics for M12. Source data are available online for this figure.
